# Building an adaptive brain across development: targets for neurorehabilitation must begin in infancy

**DOI:** 10.3389/fnbeh.2015.00232

**Published:** 2015-09-11

**Authors:** Jamie O. Edgin, Caron A. C. Clark, Esha Massand, Annette Karmiloff-Smith

**Affiliations:** ^1^Department of Psychology, University of ArizonaTucson, AZ, USA; ^2^Sonoran University Center for Excellence in Developmental DisabilitiesTucson, AZ, USA; ^3^Centre for Brain and Cognitive Development, Birkbeck, University of LondonLondon, UK

**Keywords:** Down syndrome, rehabilitation, treatment, brain development, connectivity, language, hippocampus, cerebellum

## Abstract

Much progress has been made toward behavioral and pharmacological intervention in intellectual disability, which was once thought too difficult to treat. Down syndrome (DS) research has shown rapid advances, and clinical trials are currently underway, with more on the horizon. Here, we review the literature on the emergent profile of cognitive development in DS, emphasizing that treatment approaches must consider how some “end state” impairments, such as language deficits, may develop from early alterations in neural systems beginning in infancy. Specifically, we highlight evidence suggesting that there are pre- and early postnatal alterations in brain structure and function in DS, resulting in disturbed network function across development. We stress that these early alterations are likely amplified by Alzheimer’s disease (AD) progression and poor sleep. Focusing on three network hubs (prefrontal cortex, hippocampus, and cerebellum), we discuss how these regions may relate to evolving deficits in cognitive function in individuals with DS, and to their language profile in particular.

## Introduction

It was not so long ago that having a neurodevelopmental disorder like Down syndrome (DS) or Fragile × syndrome (FXS) was a recipe for cognitive difficulties often deemed insurmountable. By contrast, the past two decades have offered much promise for the development of treatments for the cognitive dysfunction faced by individuals with such intellectual disabilities. Training programs have focused on processes like attention, memory and executive control (Conners et al., 2008; Bennett et al., 2013; Kirk et al., 2015). Investigations of pharmacological therapies have targeted specific cognitive skills such as the attention profile in FXS or memory processes in DS, tested via the use of animal models (Huber et al., 2002; Braudeau et al., 2011; De la Torre et al., 2014; Deidda et al., 2015). Intervention has also focused on content domains like language, demonstrating increases in spoken vocabulary with high frequency intervention (although this increase was less than the benefit achieved in non-DS groups; Yoder et al., 2014). While these studies are newly emerging and no single intervention has fully alleviated linguistic or cognitive deficits in humans with intellectual disability, these various successes have led to an increased awareness of the potential for successful intervention in a syndrome once thought too difficult to treat. In this sense, the last 10 years of work have provided a “proof of concept” for intellectual disability neurotherapeutics which has redirected the field, providing evidence that it may be feasible to alleviate some of the cognitive difficulties associated with syndromes such as DS, on which we focus in this article.

In the past, DS (or Trisomy 21) was often simply used as a control group for studies focused on other syndromes. With recent changes in the field, however, DS has moved into the spotlight as a model condition for exploring novel interventions and, due to initial successes, DS researchers are now actively pursuing behavioral and biomedical treatments targeting this group. However, because resources are limited, we believe that the time has come to specifically reflect on which are the most effective strategies for neurocognitive intervention in DS. In our view, to assess intervention success, the field must take a more dynamic, truly developmental approach, recognizing that the “end state” of the DS neurocognitive phenotype is *emergent* across developmental time. According to the neuroconstructivist view (Karmiloff-Smith, 1998; Mareschal et al., 2007; Karmiloff-Smith et al., 2012), cognitive-level differences in older children and adults with neurodevelopmental disorders must be traced back to their more basic precursors in infancy and early childhood. Given the dynamic interplay of cognitive systems, particularly early in development prior to specialization of function, deficits in one domain may have antecedents in several other initially interrelated domains (Karmiloff-Smith, 1998; Karmiloff-Smith et al., 2012) that are traditionally considered separate or distinct in the end-state, such as face processing, space processing, number and language. Fundamentally, this means that modifying a phenotype, such as one involving language deficits, requires an in-depth understanding of how that behavior evolved over time, pinpointing its important precursors and relations to diverse cognitive systems. Based on such evidence, intervention may require treating a different set of syndrome-specific deficits at earlier points in development (e.g., treating attention to indirectly treat language; treating saccadic eye-movement planning to indirectly treat number discrimination, etc.; see discussion in Karmiloff-Smith et al., 2012).

In our discussion of the dynamic interactions between neural systems across development, we focus on the language phenotype in DS as a clinical end-point, because language is frequently noted as the most striking deficit in this group, with sustained negative implications for quality of life and day-to-day interactions (Abbeduto et al., 2007). Language is even less developed in children with DS than would be expected given their mental age in other domains (Miller and Sedley, 1995; Boudreau and Chapman, 2000; Ypsilanti et al., 2005). Since language is impaired, a natural, direct target for intervention would be language training, since this is an area of great concern for parents or caregivers of children with DS, and a function that likely would be considered an important treatment outcome by the FDA and other public policy makers. However, we will argue that more *indirect* intervention strategies need to be developed that target the specific neural and cognitive roots of language that may be disrupted by DS very early during the pre-linguistic period.

Further, given our current knowledge of brain development in DS, we posit that interventions should aim to influence global neural organization in ways that help to normalize patterns of connectivity and establish more mature brain networks, again starting as early as possible in infancy. Rapid breakthroughs in neuroscience have emphasized the gradual specialization and refinement of neural networks and cortical hubs as the hallmark of efficient, flexible adult cognition (e.g., Buckner et al., 2009). This implies that clinical endpoints for intervention should not be limited to specialized brain regions or domain-specific systems, but instead must translate to changes at the level of network organization and efficiency. Finally, because cognition evolves across time, we emphasize that it is likely that the most effective treatments will not necessarily display their effects immediately, but only over time, perhaps even years later, as children’s brains develop as a function of processing increasingly complex environments. As a consequence, we might easily be misled about a treatment’s efficacy if, in order to gather metrics of success, we were to focus solely on immediate or short-term outcomes. Indeed, what we know about the long-term importance of patterns established during early brain development suggests that such early neural changes may dictate not only how cognitive function develops across childhood, but also how individuals respond to the aging process as adults. In the case of DS, that aging process often includes early onset AD (i.e., over 50% after age 50 years; Zigman and Lott, 2007). What follows, then, is that the clues to supporting healthy aging in DS in the fourth and fifth decades of life may, paradoxically, be rooted in childhood or infancy (Karmiloff-Smith, in press). Given that recent evidence has demonstrated differences in brain development associated with risk for AD (APOE e4 allele) in infants and children with DS (Dean et al., 2014), more emphasis should be placed on the life-span progression to AD in both typical and DS-associated decline, beginning in infancy.

## The Neural Phenotype of Down Syndrome Starts *In Utero*

Individuals with DS present with widespread differences in brain structure and function, which manifest in altered regional specializations as well as deficits in long-range neural connectivity and integration. These differences start during fetal development and continue across the lifespan. But, to set the stage, let us first briefly examine neural development in typically developing brains.

A healthy adult brain engages in processes of both information segregation and integration, resulting in efficient, flexible, cognitive processing (Sporns et al., 2000). Developmental cognitive neuroscience studies have suggested that specialized cognitive processes, such as face processing in the fusiform gyrus, become increasingly lateralized and localized to specific neural regions with time and experience (Johnson, 2001; although see Golarai et al., 2010). Such neural specialization of function may not occur in atypically developing brains, even when they display proficiency at the behavioral level (Karmiloff-Smith, 1998; D’Souza and Karmiloff-Smith, 2011). Concurrent and interactive with progressive regional specialization in healthy brains is the development of functional networks that allow for automaticity, indexing, and sustained or modulated patterns of neural firing. Recent investigations of functional brain networks in typically-developing children and adolescents have pointed to a developmental pattern of increasing segregation between close regions, coupled with strengthened correlations between key long-range connections (Dosenbach et al., 2007; Fair et al., 2008). This process allows for the differentiation of several networks important for cognitive processing, including task-control networks (“frontal-parietal” and “cingulo-opercular”), resting state and memory networks (“the default mode network”), and a cerebellar network that is functionally connected to the task-control networks. While much of the axonal wiring of these connections likely is established by 9 months of age in typical development, the efficient co-activation of hubs in these networks continues to develop across childhood and into adolescence, and may be related to increases in myelination and synaptic remodeling (Kelly et al., 2009; Gao et al., 2011; Uddin et al., 2011). Refinement of these cortical networks involves a series of progressive and regressive events, making the tracking of the nature of atypical brain development in relation to typical trajectories essential for interpreting differences in brain structure or functional connectivity (Karmiloff-Smith, 2010).

In this article, we discuss the development of brain networks in DS, focusing on some core regions within these networks, including the hippocampus, prefrontal cortex, and cerebellum. Alterations in the structure and function of these regions have been established from neurological data in humans with DS and in animal models of the syndrome (Baxter et al., 2000; Das and Reeves, 2011; Edgin et al., 2012; Fernandez and Reeves, 2015) as well as through neuropsychological investigations (Frith and Frith, 1974; Jarrold et al., 1999; Pennington et al., 2003; Vicari and Carlesimo, 2006). Our article highlights the cascading impacts on development that may arise from atypical processing in these hubs and their connections, and concludes that important end-state targets for intervention (i.e., language) could be affected by the very early wiring and tuning of networks comprising these processing regions.

There is also evidence that the brain of individuals with DS is already aberrant prior to birth and evolves to exhibit deficits in both information segregation and the formation of efficient local representational capacity, as well as long-range connectivity. Just prior to or after birth, there are global disruptions in neurogenesis, synaptogenesis and myelination (Schmidt-Sidor et al., 1990). Reduced cell number is evident in the hippocampus and surrounding cortical regions as early as 21 weeks gestation (Guidi et al., 2008). There are increased levels of amyloid-β deposition prior to birth that continue to burden neural development across the lifespan (Busciglio and Yankner, 1995; Bahn et al., 2002; Lott et al., 2006). Ultimately, together with the formation of neurofibrillary tangles (Murray et al., 2015), this amyloid-β burden leads progressively to the transition to an AD diagnosis by middle adulthood in a majority of individuals with DS.

Structural imaging studies in older children and young adults with DS have often shown reductions in the volumes of later-developing neural structures, including the frontal lobe, hippocampus, cingulate cortex, and cerebellum. Myelination between regions also is reduced, with poor development of the white matter pathways between the frontal cortex and posterior regions, including the parietal and temporal cortices (Powell et al., 2014). Very few functional neuroimaging studies have been conducted in DS, but the available data suggest that the brains of individuals with DS may have altered functional organization, marked by over-connectivity in local functional circuits (Anderson et al., 2013; Vega et al., 2015) and deviations in the spatial distribution of neural activation, in comparison to typical activity patterns.

The regional specialization of language has been examined in two separate studies. Losin et al. (2009) used a passive story-listening paradigm (contrasting forward and backward speech) in young adults with DS in comparison to chronologically age-matched controls. While the controls showed activation in classic receptive language areas, the group with DS activated a different pattern of regions in response to the speech, including greater activation in parietal cortex. Morever, unlike in the control group, neural activation patterns in the group with DS did not differ for the forward and backward conditions, showing not only that language was processed in different regions, but also that these regions had not become specialized for meaningful vs. non-meaningful speech. A separate study by Jacola et al. (2013) revealed that individuals with DS showed greater activation in the midline regions of the frontal and cingulate cortex when listening to stories as compared to tones, suggesting a need in the group with DS to recruit greater cognitive resources to process the story. Moreover, this altered functional organization is not limited to language, because a semantic classification task for objects also yielded a pattern of brain regions differing in their spatial distribution and extent of activation (Jacola et al., 2011). Adults with DS showed activation in the middle and dorsal frontal cortex relative to an age-matched control group.

Three published studies have examined cross-regional brain connectivity in young adults with DS and each has pointed to a pattern of over-connectivity in local networks and under-connectivity of long-range connections, particularly those involved in the dorsal executive systems (i.e., dorsal prefrontal cortex). Pujol et al. (2014) used fMRI to examine functional connectivity in 20 adults with DS compared to chronologically aged-matched controls. Based on whole-brain and seed regional connectivity analyses, this study demonstrated a greater degree of connectivity in short-range connections in individuals with DS, including those in the anterior temporal lobes and amygdala, coupled with reduced connectivity in certain long-range connections, including reductions in executive network connections between the dorsal PFC, ACC and the posterior insula, circuits that have consistently been associated with executive control processes. This reduction in long-range functional connectivity was significantly and highly correlated with communication skills as assessed by parent report on an adaptive behavior assessment (i.e., the ABAS-II). Correlations with individually administered measures of verbal and nonverbal IQ were not reported, however, so it is difficult to assess the specificity of these effects.

Anderson et al. (2013) also noted variations in functional brain connectivity in adults with DS, with their analyses indicating increased local network synchrony that was idiosyncratic and disorganized across participants with DS relative to typically-developing adolescents. Interestingly, levels of anti-correlation (i.e., when activation in one area increases, activation in another decreases) between functional neural networks were lower than in the control group, a finding that was replicated by Vega et al. (2015). Given that anti-correlation generally is used as a marker of specialization and differentiation of neural networks, these findings suggest more diffuse, less organized network connectivity in the brains of individuals with DS. As in Anderson et al. (2013), Pujol et al. (2014) also found that a subset of long-range functional connections was reduced in strength. On the basis of graph theory analysis, a method for modeling pairwise connections between regions, functional connectivity within the DS group was characterized by local, as opposed to long-range networks. Moreover, the posterior hubs in the default network were absent and the attention network was not developed in the DS group. In concert with Anderson’s findings, an electroencephalography (EEG) resting state analysis (Ahmadlou et al., 2013) revealed absent small-world organization in DS in the alpha- and theta-band ranges, with networks displaying more random organization.

A study examining functional connectivity using Near Infrared Spectroscopy (fNIRS) in groups of term-age infants with DS, infants born premature (<34 weeks), and a healthy, full-term control group showed that short-range connections were strong and equally developed in the preterm and full-term infants at term, but that term-age infants with DS showed reduced connectivity in these short-range connections (Homae et al., 2010). Long-range connections were not yet fully developed in any group, in keeping with findings that long-range connections only appear around 3 months postnatal development and increase thereafter. These findings indicate that short-range networks are less co-active in DS in early infancy, which is the opposite of findings in adults, but could reflect an early developing imbalance in the refinement of these networks. In total, the data suggest that the brains of individuals with DS lack the organizational structure that allows for the efficiency and flexibility found in the typically developing adult brain, and that the evolution of these differences needs to be examined across development to understand how to support healthy brain development in this group. Specifically, individuals with DS typically have immature and disorganized networks, reflecting inadequate segregation of functional regions as well as reduced long-range communication. However, if we are to utilize findings from connectivity or task-based neuroimaging in adults to determine intervention efficacy, these outcomes must first be better understood by charting differences in brain function in DS across developmental time, beginning in infancy.

Given these patterns of altered structural and functional networks in the brain, it is no surprise that language is impaired in those with DS, because this complex set of skills requires flexible interactions across multiple neural systems as well as fine-tuned local representations. Children with DS exhibit particular difficulty with expressive vocabulary as well as with the development of morphological and syntactic complexity (Chapman, 1997; Singer Harris et al., 1997; Mervis and Robinson, 2000; Fidler, 2005). In the following sections, we discuss the language profile of DS in relation to neural systems interactions that might influence these outcomes. To exemplify the neuroconstructivist perspective (Karmiloff-Smith, 1998), we frame our discussion around three neural systems of known vulnerability in DS that usually are not considered when discussing the neural underpinnings to language: the hippocampal complex, the prefrontal “executive control” system, and the cerebellum (Nadel, 2003). Only by tracking development in infancy, in relation to these neural systems and their connections, will we gain an understanding of which treatment route(s) for enhancing language acquisition may be the most effective. We now turn to these three brain circuits and their potential role in language development and delay in DS.

## The Hippocampus, Memory and Language

The hippocampus is a complex and integrative circuit with an extended developmental trajectory. In typically-developing infants, the region may have some mature structural and functional properties (i.e., intrinsic oscillations) as early as birth. Accounts of early memory formation have been documented (Mullally and Maguire, 2014), but patterns of integration of the hippocampus with other regions are still being developed across early childhood. Some researchers suggest that hippocampal structure and function continues to be modified even into adulthood (Ghetti and Bunge, 2012; Demaster and Ghetti, 2013). The hippocampus is a hub in the default mode network, a resting state network including the medial prefrontal cortex, posterior cingulate, precuneus, and the parietal cortex; this network is often associated with offline, or task-independent, processing and episodic memory (Buckner et al., 2008). Examinations of default network connectivity in typical development suggest that the hippocampus doesn’t show mature activation within this network until 2 years of age, a time frame that corresponds with a number of behavioral developments in memory (Gao et al., 2009; Olson and Newcombe, 2014). At 18–24 months, children can remember the spatial-temporal context of events and show flexibility in their memory, remembering items independently from their original learning context (Bauer et al., 1998; Robinson and Pascalis, 2004). These properties are hallmarks of mature hippocampal function, as the circuit serves as a spatial and temporal index for distributed cortical representations.

The hippocampus has been the focal point for many interventions in DS, as it has been shown to be altered in pre- and post-natal human development as well as in animal models of the disorder (Nadel, 2003). It has been repeatedly posited as the primary altered brain region leading to specific neuropsychological deficits in memory and learning (Pennington et al., 2003; Lavenex et al., 2015). Based on intervention successes in animal models, including therapies modulating excitatory-inhibitory balance, many current or proposed pharmacotherapies focus on altering the function of the hippocampus (Fernandez et al., 2007; Deidda et al., 2015). While most of the therapies targeting this region have emphasized its importance for alleviating memory deficits, there is mounting evidence regarding the additional role of the hippocampus in language learning and development, the focus of this article.

Many of the functions of hippocampus might indeed support language development. It is well-established that the hippocampus indexes arbitrary relations (e.g., association between nouns and objects) and may serve to help support these fragile associations until they can be replayed, and subsequently strengthened, over periods of sleep, a topic to which we will return in some detail (Eichenbaum et al., 1994; Paller, 1997; Mayes et al., 2007). The role of the hippocampus in learning new words, which are in most instances arbitrarily associated with their referents, has been studied extensively in typical development as well as in brain-damaged adults who had developed typically until their brain insult (Breitenstein et al., 2005; Warren and Duff, 2014). While data from patients with early focal lesions to hippocampus have suggested adequate semantic and vocabulary learning, a closer examination of their learning process has actually revealed that novel fact learning is more difficult, requiring many more repetitions than in healthy controls (Gardiner et al., 2008). What about hippocampal function in DS? Studies have suggested that individuals with DS and animal models of the condition have poor memory consolidation over long-term delays (Wishart, 1993; Smith et al., 2014). Accordingly, learning curves for vocabulary acquisition are shallow for individuals with DS, who show consistent impairments in expressive vocabulary even in comparison to other intellectual disability syndromes (Mervis and Robinson, 2000; Yoder et al., 2014).

Sleep plays a particularly important role in hippocampal memory consolidation, especially in preschool children (Hill et al., 2007; Ashworth et al., 2013). It thus is likely to affect vocabulary development, and potentially language production (Gómez and Edgin, 2015; Henderson et al., 2012). While sleep problems are common to many neurodevelopmental disorders, they are particularly pronounced in DS (Ashworth et al., 2013), with difficulties ranging from insomnias (Breslin et al., 2011), to initiating/maintaining sleep as well as excessive daytime sleepiness (Cotton and Richdale, 2006; Carter et al., 2009), to physiological problems comprising a wide spectrum of sleep-related breathing abnormalities. The reduction in sleep quality for both children and adults with DS has important implications for physical, social, and cognitive performance (Fernandez and Edgin, 2013), as well as for executive control (Chen et al., 2013). Poor sleep quality may also translate into some of the everyday difficulties experienced by individuals with DS, including daytime sleepiness, irritability, hyperactivity and impulsivity (Fallone et al., 2001).

In fact, at least 30–50% of children and adults with DS experience some form of sleep disturbance, particularly sleep fragmentation and obstructive sleep apnea, where the upper airway is obstructed during sleep, resulting in intermittent hypoxia (Owens et al., 2000; Pegg, 2006; Waldman et al., 2009; Ashworth et al., 2013). Sleep apnea is a state that limits the time spent in the deepest stages of sleep (i.e., non rapid eye-movement; non-REM periods) and a sleep state that seems to be particularly important for memory consolidation, including the integration of word knowledge. This is because it is during deep sleep that the hippocampus replays memories through a series of neurophysiological events [e.g., sharp wave ripples and associated sleep spindles, brief periods of high frequency oscillations (11–16 Hz) present in non-REM; Schabus et al., 2004]. Indeed, EEG studies of sleep in individuals with DS, in line with mouse-model studies of DS (Colas et al., 2008), demonstrate increased stage-1 sleep and reduced stage-2 non-REM sleep in this population (Miano et al., 2008). In typically developing individuals, stage 1 sleep occurs between sleep and wakefulness, and is characterized by active muscular and motor activity. Although there is a decreased awareness of sensory stimuli during this stage, individuals may not subjectively perceive this as sleep. For individuals with DS, it is this stage of sleep that is increased. On the other hand, sleep spindles, which are prominent in stage-2 sleep, are reduced from birth in DS compared to typically developing infants (Ellingson and Peters, 1980). In typically developing individuals, sleep spindles have been associated with better procedural and declarative memory, as well as the integration of new memories and existing knowledge (Tamminen et al., 2010). All in all, chronic sleep difficulties in individuals with DS are likely to have profound effects on word learning by curtailing the opportunity for neural replay that is modulated by the hippocampal system. Indeed, it has been shown that sleep disruption correlates with language development in toddlers and school-age children with DS (Breslin et al., 2014; Edgin et al., in press).

It is worth noting that changes in sleep patterns have been identified in the typically developing population some 10 or more years prior to the onset of Alzheimer’s symptomatology (Landry and Liu-Ambrose, 2014; Spira et al., 2014), and recent evidence suggests bi-directional causal links between sleep disturbance and the development of Alzheimer’s associated neuropathology in animal models (Tabuchi et al., 2015). Therefore, the sleep disturbances prevalent in DS, together with the over-expression of the APP gene on chromosome 21, may be mechanisms contributing to the rate of progression of AD in this population (Fernandez and Edgin, 2013). While further longitudinal studies are required in order to track more fully the progression of sleep architecture over developmental time, it is clear that the role of the hippocampus in memory and language learning, together with its disruption by sleep problems, cannot be ignored.

While the hippocampus is involved in the consolidation of vocabulary, the region also mediates representational flexibility and temporal coding that could support the on-line planning and use of language. Moreover, while it has often been maintained that H.M., the most studied adult patient with hippocampal amnesia, had preserved language function after his surgery, evidenced by a stable verbal IQ, some linguistic impairments were in fact subsequently reported, including deficits on complex language tasks and measures of verbal fluency (Corkin, 1984). It is difficult to attribute these deficits to the hippocampus proper because of the extent of H.M.’s damage to the surrounding cortex. Further studies with patients with isolated damage to the hippocampus have also reported difficulties in creative language use and flexible discourse (reviewed in Duff and Brown-Schmidt, 2012), and studies of adult patients with aphasia have also demonstrated an important role of the intact hippocampus for language recovery after stroke (Meinzer et al., 2010). Finally, functional neuroimaging studies have shown the hippocampus to be active during implicit statistical learning in adults, a mechanism often considered fundamental for grammar learning in infants (Gómez and Gerken, 1999; Schapiro et al., 2014).

Likewise, studies conducted with children with early left hemisphere lesions have pinpointed the potential role of the hippocampus as a driver of language lateralization (Liegeois et al., 2004). Indeed, in a study by Liegeois et al. (2004) early lesions to the usual left-hemisphere language areas (e.g., Broca’s area specifically) did not cause reorganization of language into the right hemisphere, but children with lesions specifically to the left hippocampus did show right localized and bilateral activation during a word generation task. Together with the adult patient data, these findings yet again highlight an important role for the hippocampus in contributing to the tuning of the neural networks for language.

Much remains to be explored about the role of the hippocampus in language function, but the above evidence certainly raises the likelihood that this brain structure contributes in some capacity to language acquisition across development and to the marked delays in language acquisition in individuals with DS. While it appears that the hippocampus is important for some higher-level aspects of language, it is unclear from current data when these links would first be established. The early developing functions of the hippocampus are rarely studied in humans, as it is hard to examine the function of this deep region in infant brains. Recent work on typical development from Gómez and Edgin (2015) and Edgin et al. (2014) has emphasized the fact that the role of the hippocampus in associative, flexible learning takes developmental time, gradually becoming strengthened over the childhood period. In line with this view, it is possible that *early* language development may be supported by extra-hippocampal mechanisms, while *later* emerging language capacities benefit from the flexibility afforded by the hippocampus. If Gómez and Edgin’s hypothesis regarding the late recruitment of the hippocampus for language development is correct, then early treatments supporting these networks might not yield *immediate* positive benefits on language, but may only become evident after hippocampal structures have gained full functionality and network integration (beginning at 24 months in typical development; Gao et al., 2009). In the case of those with DS, in which hippocampal development is clearly disrupted, assessing the efficacy of hippocampal circuit intervention may only be possible at an even later stage of development.

## The Prefrontal Cortex, Executive Control Networks and Language

While often considered late-developing, the networks for executive control (including prefrontal cortex, PFC) turn out to be partially active already in typically developing infants and may actually play an organizing role in cortical development (Johnson, 2012). EEG coherence studies in very young typically-developing infants have suggested that frontal activity may serve as an “organizer” of posterior activity, with frontal EEG power in infancy predicting subsequent individual performance on executive tasks at preschool age (Kraybill and Bell, 2013). In a seminal study, Dehaene-Lambertz et al. (2002) found that frontal cortex was already active in typically-developing 3-month-olds while listening to forward vs. backward speech. More recently, this French group has shown that in preterm infants, the inferior frontal cortex may assist with speech sound discrimination (i.e., phoneme and talker) prior to term age at a time when neural migration is not even fully complete (Mahmoudzadeh et al., 2013). Taken together, these findings suggest that prenatal auditory experience plays an important role in the establishment of language network architecture and that the PFC may be involved in discriminating language inputs very early in typical development.

In typically-developing children, executive control develops very rapidly during the preschool period, with more gradual improvements evident through late adolescence (Best et al., 2009; Garon et al., 2008). These advancements appear to be supported by a progressive honing of the neural circuitry underlying executive control, including the frontal-parietal, dorsal-anterior and cingulo-opercular loops. The overproduction of neural spines in the prefrontal cortex is greater than in other neural regions, while synaptic pruning in this region proceeds very slowly through childhood and adolescence, providing an extended window for experience-dependent plasticity (Ferguson and Gao, 2015). Functional neural imaging studies of typically-developing cohorts indicate that the frontal-parietal control network becomes increasingly inversely correlated with the default-mode network through the course of middle childhood, with the degree of anti-correlation predicting individual differences in general cognitive performance (Gao et al., 2009; Sherman et al., 2014). Similarly, graph theory analyses show reductions in the connections between the fronto-parietal executive network and the cingulo-opurcular salience network over the course of middle childhood, concomitant with an age-related strengthening of the connections within these networks (Dosenbach et al., 2007). These changes in functional connectivity in typical development may reflect the progressive myelination of reciprocal tracts between the prefrontal cortex and other areas of the brain, including circuits to and from the limbic regions, hippocampus and striatum (Nagy et al., 2004).

Studies of typically-developing preschool and school-age children also highlight the developmental interdependency of executive control and of a number of developmental outcomes, including language, with many of these relations likely being bidirectional in nature. Inhibitory control, for example, may provide a buffer for expressive language planning, while working memory theoretically affords the online maintenance of language inputs for processing and integration (Barkley, 1997). Executive control also has been shown to predict the development of narrative production, suggesting that it plays a fundamental role in the organization of language outputs (Friend and Bates, 2014). Any intervention to address language delays in individuals with DS will therefore benefit from the consideration of executive control networks which, as we now illustrate, are significantly atypical in DS.

While data on patterns of frontal brain connectivity are scarce in young children with DS, there is consistent evidence from adult studies to suggest that frontal volumes are selectively reduced, and that both functional and structural connectivity between the frontal cortex and the rest of the brain is altered. Data from adults with DS suggest that the fronto-parietal control network is not as clearly differentiated from the default mode network as in healthy controls (Anderson et al., 2013; Powell et al., 2014; Vega et al., 2015). Individuals with DS also show behavioral impairments on executive tasks tapping these control networks, although the degree of impairment is variable both within the DS population and across different types of tasks and age groups. Toddlers with DS show difficulties with visual sustained attention (Brown et al., 2003), although there is some suggestion that other aspects of *early* executive control, such as saccade planning and inhibitory control, may be relative strengths at this young age (Brown et al., 2003; Karmiloff-Smith et al., 2012; Roberts and Richmond, 2014). Studies conducted with older children and adolescents have generally reported pronounced deficits in verbal short-term and working memory, coupled with relative proficiency in spatial short-term memory, but impaired spatial working memory (Jarrold et al., 1999; Lanfranchi et al., 2004; Baddeley and Jarrold, 2007; Duarte et al., 2011; Yang et al., 2014). With respect to other aspects of executive control, findings in school-age children and adolescents have been mixed; some studies have reported global difficulties across multiple executive domains relative to verbal age-matched control groups (Lanfranchi et al., 2010; Borella et al., 2013). Additionally, others have reported deficits in set-shifting and selective attention (Rowe et al., 2006; Scerif and Steele, 2011; Breckenridge et al., 2013; Carney et al., 2013), with yet others finding no executive deficits beyond what would be predicted based on general cognitive performance (Pennington et al., 2003). Importantly, there is evidence that executive difficulties in DS may increase with age, particularly of course with the onset of Alzheimer’s dementia (Nelson et al., 2005; Ball et al., 2008).

The uneven profile of executive impairments in DS has clear implications for intervention and resilience, especially in the light of suggestions that executive control may help to compensate for poor functioning in other domains (Halperin and Schulz, 2006; Shaw et al., 2006; Johnson, 2012). On the one hand, in individuals with DS poor network connectivity between the PFC and other neural regions may limit the potential for plasticity and diminish the ability of prefrontal regions to coordinate and modulate sensory and semantic inputs. In particular, verbal working memory deficits in DS are likely to have cascading implications for language comprehension and everyday social interactions. Difficulties with selective attention and set-shifting may also place constraints on the amount of linguistic information that individuals with DS are able to process. On the other hand, relative strengths in at least some areas of executive control (e.g., spatial short-term memory) may help to “bootstrap” language by offering alternative processing mechanisms and management strategies. Individuals with aphasia, for example, show activation of executive control networks to a greater extent than healthy controls during normal speech (Brownsett et al., 2014), and there is evidence that executive training may facilitate recovery from aphasia (Seniów et al., 2009; Lee and Moore Sohlberg, 2013). Given that executive control appears to be particularly vulnerable to aging in DS, it may be possible to encourage the development of executive skills very early in development to mitigate later cognitive decline. Although poor executive control in DS is unlikely to be the *cause* of decreased language abilities, interventions that target improvement of executive control skills or minimize the cost of poor executive control while training language, through supports that lessen working memory and attention demands, may be more effective (see Kirk et al., 2015).

In devising a strategy for considering the role of executive control in cognitive and language development, and ways to mitigate these difficulties, it is important to consider the syndrome-specific profile of DS. First, more work is needed to understand which domains of executive control can be improved via cognitive training and which domains may show little or no improvement with training in DS. It is also likely that training strategies for a population with moderate to severe cognitive impairment will need to provide more basic scaffolding than in less severe disorders like ADHD, given that multiple cognitive systems necessary for engaging with the training are also probably impaired in DS (Kirk et al., 2015). In total, intervention strategies likely to result in more mature patterns of frontal connectivity are needed, as is a better understanding of whether or not those with DS can use the compensatory resources of the frontal cortex to their advantage. Given the consistent profile of early differences in network connectivity and decreased integration of frontal cortex with posterior regions, interventions in this domain must begin as early in development as possible, starting in infancy.

## The Cerebellum and Language

There is accumulating evidence from typical development of the importance of the cerebellum for almost all aspects of cognition, including language, executive control, spatial processing, memory, and social emotional processing (O’Halloran et al., 2012; Noroozian, 2014; Highnam and Bleile, 2015). This is reflected in the detailed topography and dense feed-forward and feedback loops to and from multiple regions of the cortex via the brainstem and thalamus (Stoodley, 2012; Buckner, 2013). Resting state functional MRI studies also provide evidence for the involvement of the cerebellum in multiple functional networks, including a motor control network, a multisensory network and an executive network, with connections between the cerebellum and language regions being especially dense (Buckner, 2013; Kipping et al., 2013). Deviations or slowing in the pace of cerebellar development, therefore, are likely to have widespread implications for diverse functions.

The cerebellum has long been recognized to be critical for the smooth execution of movement, including articulatory movements important for speech and language. It is equipped with learning mechanisms based on long-term depression that allow it to modify and adapt motor schemas as a function of external feedback (Ito, 2005; Koziol and Lutz, 2013). Although initial learning of motor sequences requires extensive involvement of cortical regions, the cerebellum becomes important for the storage of these motor schemas as they become automatic or subconscious (Ito, 2005). Given its prominent role in motor coordination, deficits in the control of articulation, gait and proprioception may well relate to cerebellar compromise in individuals with DS (Mazzone et al., 2004; Carvalho and Almeida, 2009). Even in neurodevelopmental disorders such as Williams syndrome, which presents with significantly better language production than DS, individuals experience serious problems with memory for and the timing of oro-facial articulation sequences (Krishnan et al., 2013).

It is possible that the cerebellum plays an even more important role in the acquisition of new knowledge and skills in young children, given its involvement in procedural learning (Steinlin, 2007, 2008). In healthy brains, the cerebellum increases dramatically in volume through the first year of life and shows a decrease in volume beginning in middle childhood (Knickmeyer et al., 2008; Holland et al., 2014; Wierenga et al., 2014). Recent studies suggest that cerebellar disturbances during its most rapid period of development—the prenatal period and in infancy—may be the most devastating for longer-term outcomes, with broad impacts on motor control, attention and language (Riva and Giorgi, 2000; Limperopoulos et al., 2009; Brossard-Racine et al., 2015). Lesions within the cerebellum also are related to disturbances in remote regions of cortex, emphasizing the systemic implications of cerebellar disturbances on connectivity (Limperopoulos et al., 2014). It is also worth noting that, in healthy brains, there is direct connectivity between the cerebellum and inferior colliculus that bypasses auditory cortex (Coleman and Clerici, 1987).

The cerebellum theoretically acts as a repository of procedural schemas or models for how to act on the environment, particularly with respect to timing and sequencing of activity (Stoodley, 2012; Koziol and Lutz, 2013). The region appears to play an integral role in subvocal rehearsal and is especially important when demands on timing, memory and morpho-syntactic processing increase (Ackermann et al., 2007; Mariën et al., 2014). Both imaging and lesion studies indicate that the cerebellum is involved in numerous aspects of language processing, including verbal working memory, phonological processing, semantic processing, and verbal fluency (Marvel and Desmond, 2010; van den Bosch et al., 2014; Highnam and Bleile, 2015). The cerebellum also forms part of a network of regions modulating grammar and is believed to be involved in analyzing the details of speech for regularity based on grammatical rules (Caplan and Dapretto, 2001; Mariën et al., 2014). Finally, there is evidence for a strong involvement of the cerebellum in reading, where coordination of eye and voice is crucial (Mariën et al., 2014). Difficulties in all of these areas are characteristic of the language phenotype of individuals with DS.

As in other developmental disorders, such as Autism Spectrum Disorders and Fragile × syndrome, the cerebellar system is vulnerable in individuals with DS, with cerebellar volume being reduced by almost 25% relative to healthy controls (Pinter et al., 2001; Aylward et al., 2007). Histological studies indicate that aberrations in cerebellar development are probably present before birth: the cerebellum shows reduced infolding, reduced thickness in the granule layer, dramatically reduced cell production and fewer radial glia in fetuses with DS (Guidi et al., 2011). These prenatal deviations likely reflect defects in the response of precursor cells to the Sonic Hedgehog growth factor (Roper et al., 2006). Mouse models of DS also yield fewer synapses and reduced cell density, particularly of excitatory granule cells, in the cerebellum (Moldrich et al., 2007).

What are the implications for intervention with respect to cerebellar functions? Given its particular vulnerability in early childhood, its dense interconnectivity with multiple subcortical and cortical regions as well as its unique role in the timing and modulation of speech and language, the cerebellum should form a focus of treatment and intervention efforts to address language delays in DS. Notably, cerebellar involvement in AD occurs relatively late, and it has been suggested that the automatic procedural schemas stored within the cerebellum may offer an explanation for the discrepancies in performance of habitual tasks vs. memory, planning and flexibility that accompany the onset of dementia (Ito, 2005). In DS, the picture may be different, particularly if we consider this from a developmental perspective: although the onset of AD does not relate to decreases cerebellar volume (Aylward et al., 2007), disruptions to cerebellar development *early in life* may limit the potential for compensation based on well-learned procedural memories.

Therefore, interventions that target cerebellar function during its critical period of growth in infancy may have implications for the subsequent development of important adaptive skills, including language (Brunamonti et al., 2011; Schott and Holfelder, 2015). Reversal of cerebellar pathology in infancy has been accomplished in animal models (Das et al., 2013). Through the injection of a sonic hedgehog pathway agonist (SAG 1.1) at birth in the Ts65dn DS mouse model, the cerebellum was normalized in size in adulthood, an effect that resulted in improvements in learning outcomes in the model. Much remains to be explored regarding the clinical application of this treatment in humans, but the logic underlying the animal models could provide the field with a useful mechanism to explore the cerebellum’s role in the development of functional brain networks throughout the lifespan.

## Conclusion

This review illustrates how having an extra chromosome 21 in DS affects multiple neural systems that are likely to play a role in sculpting a healthy, adaptive brain to enable it to develop good language skills. The majority of the findings reviewed above highlight the existence of neural differences in DS that are already present prior to birth, together with factors like amyloid deposition and sleep disruption that progressively exacerbate the problems that individuals with DS have in compensating for these early neural differences. This leads us to the conclusion that if we are to have the greatest influence on changing neuro-cognitive outcomes, we must: (1) begin treatments as early as possible in infancy, when functional dissociations are first becoming established; (2) not necessarily train in the domain of cognitive-level deficits (e.g., language) but in their basic-level underpinnings (e.g., attention, sleep); (3) incorporate temporally distal endpoint measures (i.e., both brain and neuropsychological) that embrace the interconnected nature of cognition; (4) devote more resources to understanding early patterns of brain and behavioral development in DS and how they may drive functional outcomes; and (5) gain insight into the extent to which additional burdens from amyloid deposition and sleep disruption may keep those with DS from utilizing resources to compensate for early deficits, by targeted sleep interventions. In addition to this, we stress the fact that intervention needs to be syndrome-specific (Cornish et al., 2007, 2012), that intervention is time-dependent (Karmiloff-Smith et al., 2014; Massand and Karmiloff-Smith, 2015), and that assessing whether intervention is successful must address the question of when across developmental time intervention changes are likely to become neurally manifest.

To exemplify some of these points, we consider approaches to reading intervention in individuals with DS. From early on, children with DS display atypical trajectories in reading ability (Cardoso-Martins et al., 2009). However, visual memory and word recognition skills do not appear to be as impaired as novel-word decoding and reading comprehension, which present a particular difficulty for children with DS (Bird et al., 2000). It is of interest that this profile of reading impairment is not characteristic of developmental disorders in general but seems syndrome-specific (Steele et al., 2013). For example, Steele and collaborators found that unlike children with WS matched on mental age, the reading performance profiles of those with DS were characterized by poor phonological awareness and vocabulary but good single word reading and letter knowledge. These findings illustrate our point that it is important to consider whether the long-term benefits of treatment programs may only be observed for individuals who receive treatment that is syndrome-specific, i.e., tailored to their specific profile of strengths and weaknesses in reading ability.

Several attempts have been made to better understand which types of reading and language interventions work best and which fail for children with DS. Burgoyne et al. (2012) tested the efficacy of a reading and language treatment for 57 children with DS, which included a 40-min daily intervention targeting vocabulary, phonics and word recognition. After 20 weeks of intervention, the children with DS demonstrated improved single-word reading, letter-sound knowledge, phoneme blending and expressive vocabulary. However, the results were no longer significant at 40 weeks. Moreover, the effects did not generalize to other reading and language skills, such as expressive/receptive vocabulary, non-word reading, spelling, or expressive grammar. The findings of this study suggest that although improvements can be obtained in these interventions, these are usually short term and only observed for the skills directly taught. In other words, after most interventions, children with DS do not readily generalize their learned skills to other tasks that were not directly trained (similarly to interventions targeting memory; Conners et al., 2006). On the whole, while intervention and training studies have yielded some promising short-term results for reading and language improvements for individuals with DS, most studies have failed to find long-term effects or transfer to untrained materials.

In another example, most children with DS often first learn to read using “Look and Say” approaches, which involve learning the associations between a spoken form of a word and the whole printed word (Singh and Singh, 1986). One problem with this approach is that it does not equip the child with the skills to decode newly encountered words. As an alternative to the “Look and Say” approach, children can be taught to segment a word into sounds and blend the sounds into words (“word analysis,” Department for Education and Skills., 1998). Although this type of intervention improves the ability for individuals to “sound out” words, it has not been successful at improving non-word reading tests in DS, which serve as markers for how well they will do when encountering new reading materials (Goetz et al., 2008). Jarrold and Baddeley (1997) have argued that, because the auditory memory skills required for word analysis are weak in DS, the auditory information required to sound out the words is not available long enough for individuals with DS to complete the task.

Overall, then, these studies demonstrate that several current approaches to language and reading intervention have focused on direct training of word recognition and vocabulary. As we have illustrated throughout this article, deficits in vocabulary consolidation, verbal working memory, language planning and language analysis likely reflect underlying disruption to core neural hubs that manifests in different ways across development. For instance, disruptions to sleep likely mean that vocabulary training may quickly be lost in individuals with DS because hippocampal replay is not allowing for effective consolidation of this knowledge. Sleep interventions beginning in infancy may therefore have compounding positive implications for reading and language. Difficulties in planning and verbal short-term memory may also hinder reading comprehension and limit the efficacy of sound-blending approaches to intervention. Additional training and support for frontal and cerebellar short-term memory and planning functions may therefore provide a useful compliment to training in phonemic awareness or word recognition.

The findings reviewed here further indicate that we should devote resources to therapies with the potential to normalize, *early* in the DS developmental trajectory, patterns of functional brain connectivity. In terms of candidate therapeutic approaches, we argue that the brain of individuals with DS must be helped to maintain the right balance between excitation and inhibition in order to strengthen and synchronize long–range connections as well as to hone local networks (Cline, 2005; Buzsaki, 2006). Indeed, one predominant theory of neuronal dysfunction in DS invokes an imbalance between excitation and inhibition (Kleschevnikov et al., 2004; Fernandez and Garner, 2007), so studies modifying inhibition should also examine the extent to which such interventions drive alterations in brain connectivity across development. Treatments targeting the normalization of cerebellar function early in life are also promising and provide a tool with which to determine the influence of this structure on the development of functional networks. While researchers have explored the impacts stemming from cerebellar modification on hippocampal-dependent memory performance, the effects may actually be even broader extending, for instance, to language. Whereas this work has led to candidate mechanisms that could help to alleviate cognitive difficulties (Fernandez and Garner, 2007; Das et al., 2013), it should be noted that to date the above approaches have only been shown to be effective in animal models. Much work must be done to determine whether they will translate to humans; part of this task involves understanding the developmental time frames in which intervention could be most effective. We believe that these and other treatments, including behavioral interventions, should be executed as early as is possible in development (when deemed to be safe).

Finally, an open question is the extent to which those with DS may be able to benefit from compensatory functions afforded by executive networks and frontal cortex. While frontal cortex is often thought to enable compensation for deficits in other neural systems, it remains unclear whether those with DS will be able to benefit from the training of these processes in the same manner. Given our data showing that sleep disturbances also relate to variability in executive control, it may be the case that, without targeted sleep interventions, sleep deficits could also limit the ability of those with DS to utilize the frontal cortex to compensate for altered development in other systems.

Much remains to be explored at this time in history in which interventions for cognitive differences in DS are being implemented at a rapid pace. In our view, a lifespan perspective is critical (Edgin et al., 2012; Farran and Karmiloff-Smith, 2012), meaning that it is also crucial for the field to step back and determine in far more detail precisely how the cognitive and neural phenotypes of DS (and of other neurodevelopmental disorders) evolve, which would allow for the targeting of intervention at the neural systems level.

## Conflict of Interest Statement

Jamie Edgin is a paid consultant for F. Hoffman La Roche Ltd and Novartis. The other authors declare that the research was conducted in the absence of any commercial or financial relationships that could be construed as a potential conflict of interest.
